# Facile synthesis, crystal structure, biological evaluation, and molecular modeling studies of *N*-((4-acetyl phenyl) carbamothioyl) pivalamide as the multitarget-directed ligand

**DOI:** 10.3389/fchem.2022.992701

**Published:** 2022-09-26

**Authors:** Aamer Saeed, Syeda Abida Ejaz, Aqsa Khalid, Pervaiz Ali Channar, Mubashir Aziz, Tanveer A. Wani, Seema Zargar, Sidra Hassan, Hammad Ismail, Dania Khalid, Muhammad Zaffar Hashmi, Tuncer Hökelek, Abdullahi Tunde Aborode

**Affiliations:** ^1^ Department of Chemistry, Quaid-i-Azam University Islamabad, Islamabad, Pakistan; ^2^ Department of Pharmaceutical Chemistry, Faculty of Pharmacy, The Islamia University of Bahawalpur, Bahawalpur, Pakistan; ^3^ Department of Basic Sciences, Mathematics and Humanities, Dawood University of Engineering and Technology, Karachi, Pakistan; ^4^ Department of Pharmaceutical Chemistry, College of Pharmacy, King Saud University, Riyadh, Saudi Arabia; ^5^ Department of Biochemistry, College of Science, King Saud University, Riyadh, Saudi Arabia; ^6^ Bahawalpur College of Pharmacy, Bahawalpur Medical and Dental College, Bahawalpur, Pakistan; ^7^ Department of Biochemistry and Biotechnology, University of Gujrat, Gujrat, Pakistan; ^8^ Department of Chemistry, COMSATS University Islamabad, Islamabad, Pakistan; ^9^ Department of Physics, Faculty of Engineering, Hacettepe University, Ankara, Turkey; ^10^ Department of Chemistry, Mississippi State University, Starkville, MS, United States

**Keywords:** synthesis, DFTs, Hirshfeld, molecular docking, *in vitro*

## Abstract

The crystal structure of *N*-((4-acetylphenyl)carbamothioyl)pivalamide (3) was synthesized by inert refluxing pivaloyl isothiocyanate (2) and 4-aminoacetophenone in dry acetone. The spectroscopic characterization (^1^H-NMR, ^13^CNMR, FT-IR) and single crystal assays determined the structure of synthesized compound (3). Systematic experimental and theoretical studies were conducted to determine the molecular characteristics of the synthesized crystal. The biological examination of (3) was conducted against a variety of enzymes i.e., acetyl cholinesterase (AChE), butyl cholinesterase (BChE), alpha amylase, and urease enzyme were evaluated. The crystal exhibited approximately 85% enzyme inhibition activity against BChE and AChE, but only 73.8 % and 57.9% inhibition activity against urease and alpha amylase was observed respectively. The theoretical calculations were conducted using density functional theory studies (DFTs) with the 6–31G (d, p) basis set and B3LYP functional correlation. The Frontier molecular orbital analysis revealed that the HOMO/LUMO energy gap was smaller, which corresponds to the molecule’s reactivity. In terms of reactivity, the chemical softness value was found to be in good agreement with experimental values. In Crystal structure analysis, the intramolecular N—H•••O hydrogen bond generates a S 6) ring motif and N—H•••O interactions exist in crystal structure between the centroids of neighboring parallel aromatic (C4-C9) rings with a centroid to centroid distance of 3.9766 (7)Å. These intermolecular interactions were useful in structural stabilization. The Hirshfeld surfaces and their related two-dimensional fingerprint plots were used for thorough investigation of intermolecular interactions. According to Hirshfeld surface analysis of the crystal structure the most substantial contributions to the crystal packing are from H ••• O and H ••• N/N ••• H interactions. Molecular docking studies were conducted to evaluate the binding orientation of synthesized crystal with multiple targets. The compound exhibited stronger interactions with AChE and BChE with binding energies of -7.5 and -7.6 kcal/mol, respectively. On the basis of *in-vitro* and *in-silico* findings, it is deduced that *N*-((4-acetylphenyl)carbamothioyl)pivalamide 3) possesses reactive and potent multiple target inhibitory properties.

## 1 Introduction

**FIGURE 1:**
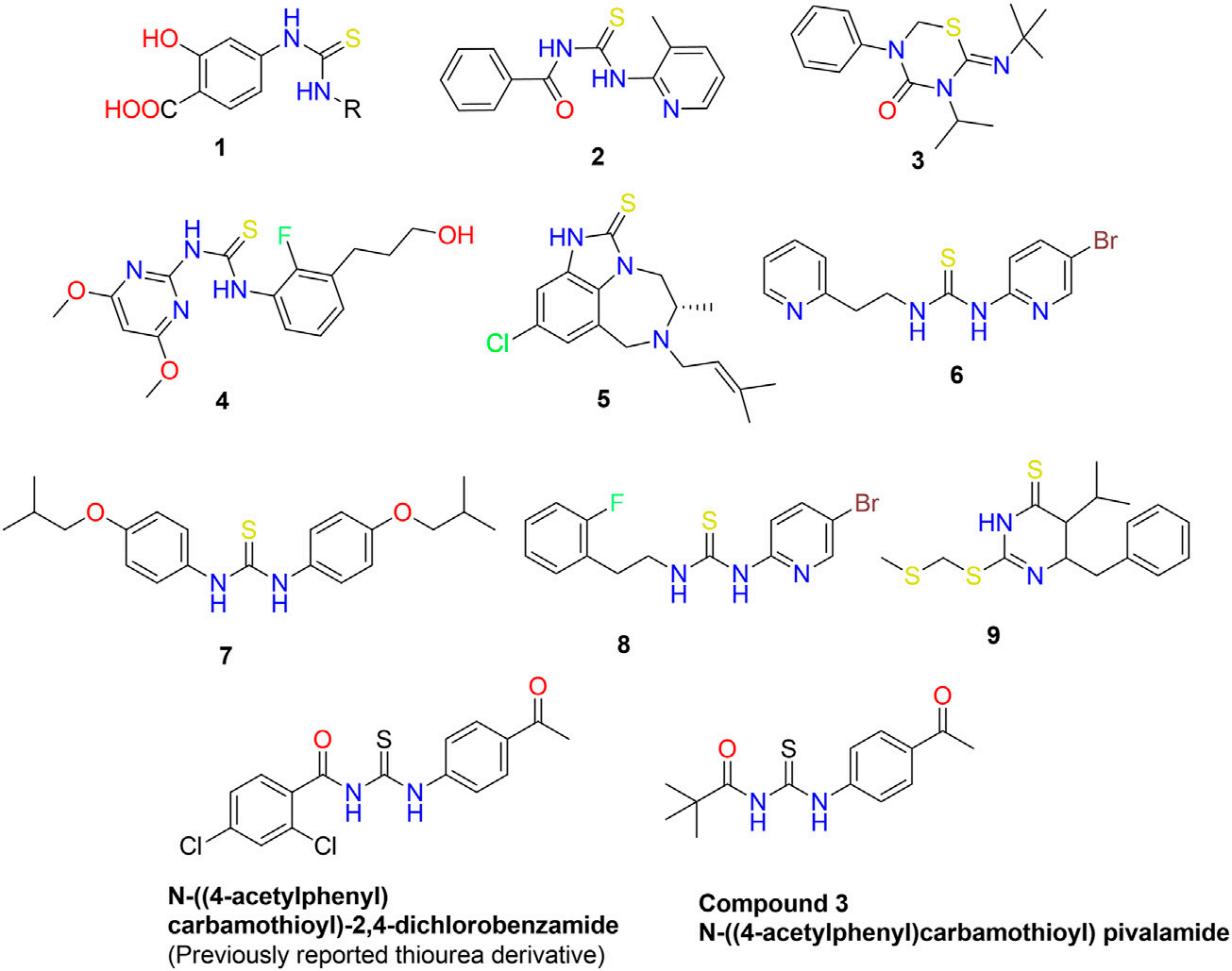
Bioactive aliphatic and cyclic thiourea derivatives 1–10 (Wright and Hallstrom, 2006; Saeed et al., 2017a; Saeed et al., 2017b; Larik et al., 2018).

Nitrogen-containing heterocyclic compounds belong to a distinct class that has contributed to several organic synthesis protocols and has a wide range of applications in the field of chemistry (Li et al., 2013). The *N*-heterocyclic compounds such as dyes, antibiotics, vitamins, and agrochemicals have depicted a wide variety of pharmacological and physiological properties (Santos et al., 2018; Kerru et al., 2019). The RNA and DNA base pairs, purines and pyrimidines, are also composed of *N*-heterocyclic molecules. The high electron density of these compounds facilitates the formation of intermolecular interactions such as van der Waal, hydrogen bonding, and pi-stacking interactions (Leeson and Springthorpe, 2007). Acyl thiourea are important nitrogen containing heterocyclic compounds that possessed diverse biological activities. In the last three decades, the synthesis and use of 1,3-disubstituted thiourea derivatives have increased significantly. Several heterocyclic compounds have been synthesized using 1-(acyl/aryl)-3-substituted thioureas as a starting material. Due to the presence of two acidic protons, one on each nitrogen atom, as well as carbonyl and thiocarbonyl groups, these are crucial precursors for the formation of numerous heterocycles. They are the ligands with a high potential for coordination because their structures contain both soft and hard donor sites. The adaptability of thiol-thione tautomerism is a result of diverse coordination modes. Sulfur and nitrogen are both electron-coordinating sites, and compounds with a thioamide structure coordinate easily. Moreover, different scientists (Wright and Hallstrom, 2006; Saeed et al., 2017a; Saeed et al., 2017b; Larik et al., 2018) studied the metal ion and ligand relationship between platinum and 1-(acyl/aroyl)-3-(alkyl) and 1-(acyl/aroyl)-3,3-(di-alkyl)-thioureas (1, 2). Acyl thiourea scaffolds are one-of-a-kind “collectors” for preferentially retaining soft metal cations in froth flotation procedures for mineral extraction. Recently, thiourea metal complexes with various metals have been synthesized and examined for environmental management as well as their luminescent properties (Saeed et al., 2014). They possess very intriguing luminescent properties and applications. Moreover, thiourea derivatives possess a wide variety of bioactivities, and biological assays for various biological applications, such as anti-tuberculosis activity (Zahra et al., 2022), have been investigated. The brown planthopper is a species of plant pest that primarily attacks rice crops. They harm plants by transmitting disease-causing viruses and feeding cell soap directly (Bruce et al., 2007). Environment friendly insecticides for brown planthoppers are derivatives of thiourea 3) (Figure 1) (Semeniuc et al., 2010). Controlling fungi is crucial because fungal diseases reduce crop yields. Recently, scientists have been searching for new molecules 4) with enhanced antifungal properties (Zaib et al., 2014). The human immunodeficiency virus (HIV) impairs a patient’s immune system and its ability to fight off disease. As a result of a compromised immune system, HIV patients are susceptible to numerous other diseases and infections, including *tuberculosis*. Therefore, it was necessary to develop a class of compounds capable of treating multiple diseases simultaneously. This innovation relieves the patient from pill burden and toxicity of HIV and *tuberculosis* treatment (Saeed et al., 2010). Anti-HIV agents include tetrahydroimidazo benzodiazepinthione derivatives 5) and Trovirdien 6) produce severe unfavorable effects. Moreover, T.B. is treated with ISOXYL (7). Additionally, D-PTB (8), F-PTB 9) and S-BABO 2) exhibited potential anti-HIV activity (Saeed et al., 2009; Saeed et al., 2017b).

Given the therapeutic relevance of the thiourea scaffold, it is necessary to examine its molecular structure, non-covalent interactions, thermodynamic and electrical characteristics in order to determine the impact of various substituents on the biological activity of the parent chemical. In this context, DFT investigations are an effective method for establishing a correlation between theoretical and experimental compound properties. These investigations offer adequate information about the compound’s molecular structure, electronic and thermodynamic characteristics. These methods have the potential to determine the molecular characteristics of a substance with precision and accuracy. (Karrouchi et al., 2020). The existence of non-covalent interactions, including hydrophobic and hydrophilic interactions, has a substantial effect on the molecular characteristics of bioactive compounds. Among these interactions, the significance of pi-stacking and hydrogen bonding interactions has been established in biological chemistry, however the significance of the other interactions remains unexplored. In order to determine the functional and structural roles of non-covalent interactions, systematic experimental and theoretical investigations were performed (Al-Otaibi et al., 2020). Comprehensive computational investigations, including x-ray analysis, Hirshfeld analysis, DFT (Noureddine et al., 2021), and molecular docking studies, were conducted in this respect. These *in-silico* studies are an effective method for assessing a compound’s biological characteristics and let to development of novel bioactive substances with unique applications in material sciences. Several docking studies have been used into the drug design and development process (Gatfaoui et al., 2020). However, it was intriguing to compare experimental and theoretical data to compare the structural aspects of (3). In this regard, biological evaluation of 3) was conducted against multiple targets. The *in-vitro* and *in-silico* studies demonstrated the unique properties of compound (3).

We have previously reported the N-((4-acetylphenyl) carbamothioyl)-2,4-dichlorobenzamide derivative (Figure 1) as a possible urease inhibitor based on experimental and computational studies (Khalid et al., 2022). The present work aims to investigate N-((4-acetylphenyl)carbamothioyl) pivalamide 3) as a multi-targeted chemical that might be exploited as a drug-like contender in the future. For this reason, the molecule was synthesized and its inhibitory ability against four separate enzymes (α-amylase, Urease, acetylcholinesterase, and butyrylcholinesterase) was determined.

## 2 Experimental

### 2.1 Materials and methods

The melting point was determined without correction using a Gallenkamp melting point device (MP-D). The infrared spectra were recorded using KBR pellets on a Shimadzu IR 460 spectrometer. The ^1^H-NMR spectra were collected in deuterated solutions using a Bruker 300 MHz spectrometer with TMS as the internal standard. A^13^C NMR spectrum was generated using a 75 MHz instrument. Chemical shift (CS) values are measured in parts per million (ppm). A GC-MS 6890N equipment from Agilent Technologies was utilized to record the mass spectrum (EI, 70 eV). On an aluminum plate precoated with silica gel, thin-layer chromatography (TLC) was performed (layer thickness 0.2 mm, HF 254, Reidalde-Haen from Merck). UV light was utilized for chromatogram visualization (254 and 260 nm).

#### 2.1.1 General method used for the synthesis of *N-((4-acetyl phenyl) carbamothioyl)* pivalamide (3)

The potassium thiocyanate (10 mmol) was dropwise added to the pivaloyl chloride (5.0 mmol) in dry acetone (20 ml) and the mixture was refluxed for three hours. The reaction mixture was then allowed to cool to room temperature. The reaction mixture was then refluxed with a solution of 4-amino acetophenone (5 mmol) in dry acetone for 24 h. TLC was employed to monitor the development of the reaction. Upon completion of the reaction, the reaction mixture was poured over crushed ice and the product thiourea 3) was precipitated, filtered, and recrystallized with ethanol.

**Figure:**
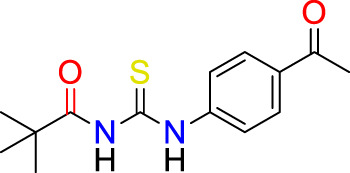


##### 2.1.1.1 *N-((4-acetyl phenyl) carbamothioyl)* pivalamide (3)

Light Green solid; M.P: 140°C; Yield: 79% R_*f*_: 0.63, Ethyl acetate: n-Hexane (4:6); FTIR (cm^−1^): 3,233 (NH Stretch), 2,955 (sp^2^ CH-Ar), 2,936 (sp^3^ CH), 1,655 (C=O), 1,525 (C=O), 1,174 (C=S); ^1^H-NMR; (300 MHz, Acetone): δ 12.95 (s, 1H, NH), 9.40 (s, 1H, NH), 8.06–7.96 (m, 4H, Ar-H),2.89 (s, 3H, CH_3_); 1.38 (s, 9H, 3*CH_3_); ^13^C-NMR (75 MHz, Acetone): δ 195.91 (C=O); 180.25 (C=S); 178.8, (C=O); 142.1, 134.6, 128.9 122.8(Ar-C); 40.1 (C), 29.7 (3*CH_3_), 25.7 (CH_3_).

### 2.2 Enzyme inhibition assay

α-amylase enzyme inhibition activity of 3) was performed using previously reported method (Shahzad et al., 2019). For experiment purpose, 0.2U of *a*-amylase enzyme solution, 10 μl starch, 10 μl of 3) with 200, 100 and 50ppm concentration was mixed in 96-well plates. All the plates were incubated at 50°C for 30 min. Then 100 µl of HCl (0.2 M) was added as stopping reagent followed by addition of 100 µl of iodine reagent (5 mM KI and 5 mM I2). The urease inhibition activity of 3) was carried out by the previously reported method (Ahmed et al., 2021). For experiment 0.1U enzyme, 10 μl of 3) with 200, 100 and 50ppm concentration, 50 μl buffer consisting of 100 mM urea, 0.01 M LiCl_2_ and 1 mM EDTA was mixed in 96-well plates. The reaction mixtures were incubated for 15 min at 37°C. After that, 50 μl of phenol reagent and 50 μl of alkali reagent were added to each well, and the plates were incubated for 50 min at 37°C. The cholinesterase inhibition assays (Larik et al., 2018) were carried out as per Ellman’s method, with a few developments (3–5). For the experiment, 25l (15 mM) substrate (acetylthiocholine iodide for acetylcholinesterase and butyrylthiochloline iodide for butyrylcholinesterase), 50l (0.1 M) sodium phosphate buffer (pH 8.0), 125l (3 mM) DTNB, and 25l of 3) with 200, 100, and 50ppm concentrations were used. The plates were then incubated for 30 min at 37°C. For the antidiabetic and cholinesterase assays, triplicates of acarbose and galantamine hydrobromide were used as positive controls. The absorbance of urease was measured at 625 nm, whereas the absorbance of the other tests was obtained at 405 nm using a microtiter plate reader (Elx-800), and percentage inhibition was determined using the formula provided.

### 2.3 X-ray crystal structure and x-ray refinement

The crystallographic data of synthesized compound 3 was collected by a diffractometer (Rigaku Oxford Diffraction Xcalibur, Eos, Gemini) equipped with Cu *K*_α_ radiation (*λ* = 1.54184 Å). The same methodology was employed as reported in our previous study (Khalid et al., 2022). The detailed methodology for crystallographic data analysis is given in supplementary data.

### 2.4 Hirshfeld surface analysis

Hirshfeld surface (HS) analysis is the most effective method for determining crystal structure interactions (Ashfaq et al., 2021). Using Crystal Explorer 17.5 (Bhola et al., 2019), the intermolecular interactions of the crystal structure of the subject molecule were seen. On the interior and exterior of the surface, the Hirshfeld surface distance from the nearest nucleus was measured and represented by D_i_ and D_e_, respectively, while D_norm_ was used to denote the normal contact distance. D_norm_ has been spelled in white, red, and blue. The CIF-formatted input file is retrievable from the supplemental materials.

### 2.5 Density function theory studies

The DFT studies were conducted to optimize the molecular geometry of the synthesized derivative (Sagaama et al., 2020a). The structural geometry was minimized to provide the steepest energy gradient at a point where no imaginary frequencies was observed. All of these theoretical calculations were performed with the Gaussian 09 W programme, B3LYP functional correlation, and 6–31G (d, p) as the basis set (Frisch et al., 2016). In addition, various output data files, including log files, were evaluated with Gauss View 6.0 (Dennington et al., 2016). The molecule underwent FMO investigation, which included HOMO/LUMO energy gap, chemical hardness, and chemical softness evaluations. In addition, polarizability and optimization energy were calculated using the same degree of theory.

### 2.6 Molecular docking methodology

Molecular docking studies are an effective method for determining the binding orientation of a ligand with a specific protein. The protein selection criterion was based on the available literature as well as the nature of enzyme source used for the *invitro* studies. This was done to link our in-silico results with the *in-vitro* analysis. The compound 3, was subjected to *in-vitro* and in-silico testing against four different targeted proteins, i.e., AChE, BChE, α-amylase, and urease. Initially, the crystallographic structures of all proteins were retrieved from the protein data bank (www.rcsb.org) using PDB IDs 2QMK, 4AC7, 7RB5, and 6EP4 respectively. All these proteins were prepared using the AutoDock suite, which included the removal of het and water molecules, the addition of polar hydrogen and kollman charges. Moreover, missing residues were also prepared. After preparation of targeted proteins, the 2D structure of Ligand was generated by using Chemdraw (Miao et al., 2015), from which it was converted to an SDF file and consequently to PDBQT (Berman et al., 2003). The ligand and receptor protein are subjected to interact freely so that the best site can be identified in the receptor protein where maximum interactions takes place. The maximum spacing is selected so that major part of the receptor can be covered in the grid box where interactions are visualized. The grid dimensions for all the receptors for all the three axis x, y and z is 26 Å with spacing of 1Å to maximize the interaction space. The grid coordinates placed at center of protein vary from protein to protein on all sides. The center x, y and z for AChE are 6.430, 39.109 and −0.694 respectively. The center for BChE at x y and z are −16.243, −32.154 and −26.738 respectively. The center for amylase are 8.493, −27.955 and 15.695 for x, y, and z respectively. The center for urease at x, y, and z are 15.994, 94.780 and 74.110 respectively. The docking was performed *via* AutoDock VINA (Trott and Olson, 2010). The results were analysed by using PyMOL (Yuan et al., 2017) where binding residues and bond lengths were noted.

## 3 Results and discussion

### 3.1 Synthesis

**SCHEME 1:**
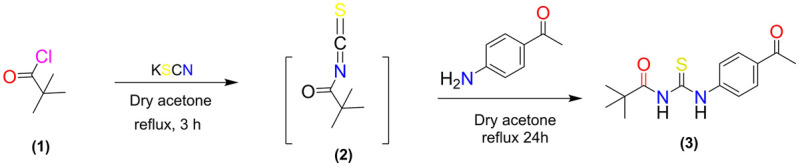
Synthetic pathway to *N*-((4-acetyl phenyl) carbamothioyl) pivalamide

A synthetic procedure for *N*-((4-acetyl phenyl) carbamothioyl) pivalamide is shown in Scheme 1. The target molecule was synthesized using pivaloyl chloride and 4-aminoacetophenone. Initially, potassium thiocyanate was used to convert pivaloyl chloride 1) to pivaloyl isothiocyanate (2). The reaction of pivaloyl isothiocyanate 2) with 4-aminoacetophenone was performed in dry acetone in an inert environment. TLC was used to monitor the reaction’s progress, using n-hexane and ethyl acetate (6:4) as mobile phase. The resulting product was recrystallized from ethanol to yield a pure title compound (3). The made derivative was looked at by using ^1^H NMR, ^13^C NMR, and FT-IR spectroscopy.

The FT-IR spectrum displayed a characteristic stretching vibration for the NH functionality at range 3,233 cm^−1^ aromatic stretch 2,955 cm^−1^ (sp^2^ CH-Ar), aliphatic (sp^3^ CH) at 2,936 cm^−1^ and C=O at 2,955 cm^−1^. The ^1^H NMR spectrum of derivative **3** showed two characteristic signals of N-H at δ 12.95 and 9.40 ppm. Two multiplet for four aromatic protons appeared at δ 8.06–7.96 ppm, singlet for nine protons of CH_3_ at δ 1.38, and a singlet for CH appeared at δ 2.89 ppm. In ^13^C NMR, three characteristic signals, one for thiocarbonyl and two for carbonyl carbons were observed. These signals appeared at δ195.91 for C=O, δ180.25 for C=S and δ178.8, ppm for another C=O). Four carbons of aromatic system observed at δ142.1–122.8ppm. Three signals for aliphatic carbons observed in the range δ 40.1–25.7 ppm.

### 3.2 Enzyme inhibition assay and molecular docking studies

**FIGURE 2:**
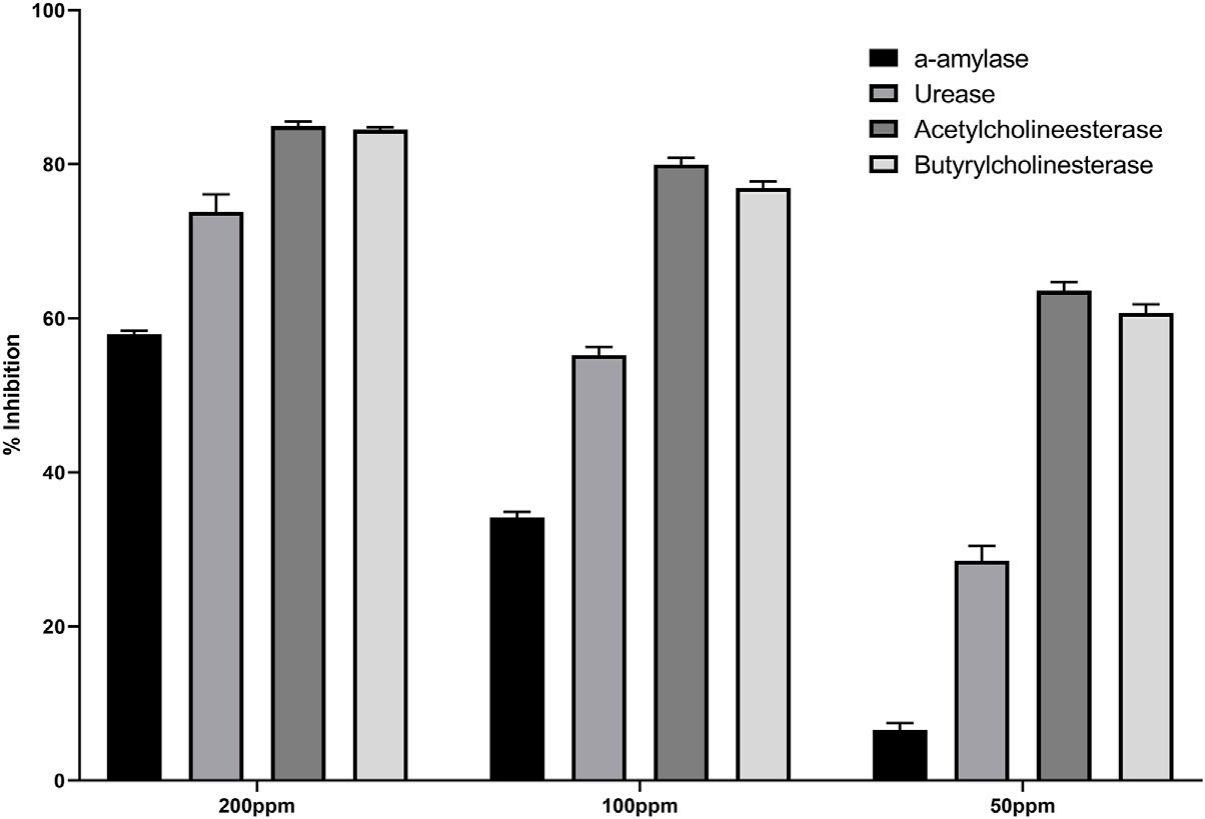
Percentage inhibition of (3) against different enzymes.

The enzyme inhibition potential of compound 3 was examined using the 96-well method, and the percentage inhibition response in a concentration-dependent manner is depicted in Figure 2. Compound 3 exhibited around 85 percent enzyme inhibition activity against butyrylcholinesterase and acetylcholinesterase, whereas its inhibitory activity against urease and alpha-amylase was 73.8 percent and 57.9 percent, respectively. Compound 3 had potential activity against AChE and BChE with IC_50_ values of 26.23 and 30.9 ppm, respectively. However, IC_50_ values against alpha amylase and urease enzymes were marginally higher (160.33 and 91.5 ppm respectively) (table 1).

%inhibition=(Absorbance of control−Absorbance of sample)/Absorbance of control×100

**TABLE 1:** Enzyme inhibition assay and Molecular docking scores.

Receptor/Assay	Enzyme inhibition IC_50_ ± SD (ppm)		Docking scores	
	Compound 3	Positive control	Binding energy (kcal/mol)	Binding residues/Bond lengths (Å)
α-amylase	160.33 ± 1.7	15.4 ± 2.3	−6.1	Q-63 = 3.0
Urease	91.5 ± 3.9	23.3 ± 0.9	−6.3	Q-460 = 3.0
Acetylcholinesterase	26.23 ± 2.7	10.9 ± 0.6	−7.5	Y-124 = 2.9, F-295 = 3.2, Y-337 = 3.1, 3.2, Y-341 = 3.1
Butyrylcholinesterase	30.9 ± 2.3	12.6 ± 2.1	−7.6	G-117 = 3.3, S-198 = 2.9

**FIGURE 3:**
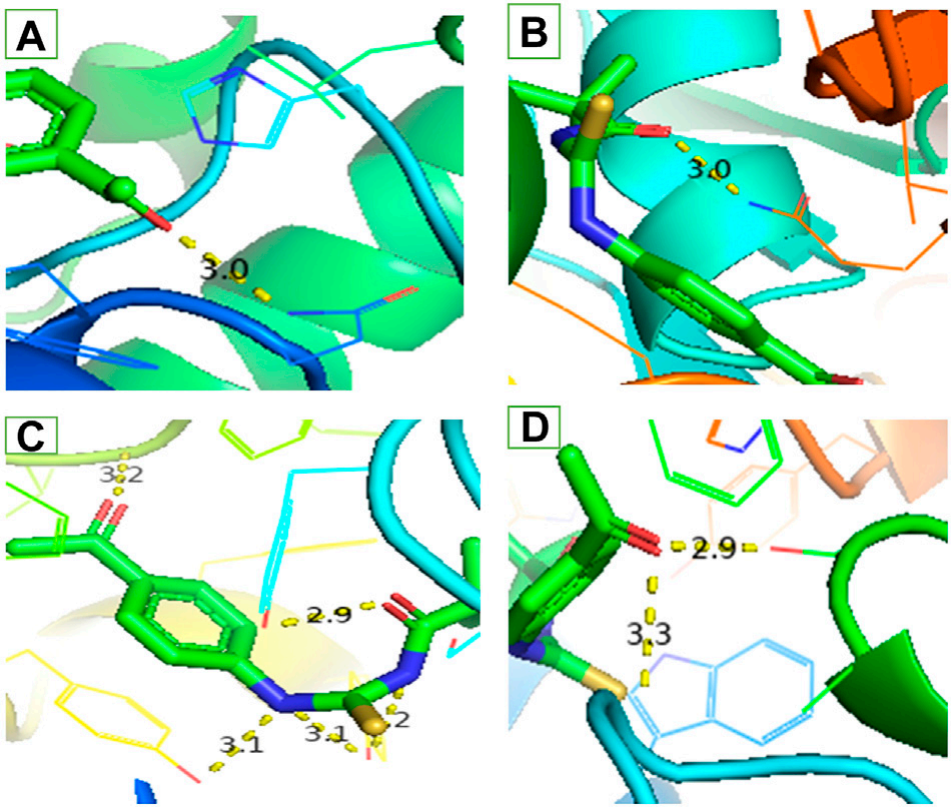
Interaction of ligand (3) **(A)** *a*-amylase **(B)** Urease **(C)** Acetylcholinesterase and **(D)** Butyrylcholinesterase.

Secondly, compound 3 has moderate activity against urease with IC_50_ value 91.5 ± 3.9 ppm. Finally, against *a*-amylase comparatively lower activity was recorded with IC_50_ value 160.33 ± 1.7 ppm. Following the experimental findings compound 3 was docked with all enzyme proteins to identify the possible mode of interactions responsible for their activities and results are summarized in Figures 2, 3 and table 1. For statistical confirmation each ligand was interacted five times with the respective protein and statistically significant results along with best interaction is presented in the manuscript.

Similar to enzyme inhibition experiments, docking results showed remarkable energies in order of BChE > AChE > Urease > alpha-amylase. Both acetylcholinesterase and butyrylcholinesterase receptors exhibited robust interactions with the ligand compound 3 at energies of −7.5 kcal/mol and −7.6 kcal/mol, respectively. Similarly, alpha-amylase and urease exhibited comparable and significant interactions with compound 3, with binding energies of −6.1 and −6.3 kcal/mol, respectively. The interactions present in docked ligand receptors include Vander Val forces, ionic and hydrogen interactions. The bond energy are low and bond lengths are less than 4 Angstroms which indicate a good interaction. These interactions take place between charged groups on ligand and different amino acid residues of the protein that are described in the Table 1. The ligand compound 3 interacted with only one residue of each alpha amylase and urease with an oxygen atom of carbonyl groups of both proteins. BChE’s two residues reacted with one oxygen atom of ligand’s carbonyl group whereas AChE’s four residues interacted with ligand where 1 residue formed interactions at two points making a total of five interactions. The two residues Y-124 and F-295 bonded with two oxygen atoms of different carbonyl groups, the remaining three bonded with hydrogen atoms as two with Y-337 and one with Y-341. This is in accordance with previous studies where morphine-protein interactions, monoamino oxidases-ligand interactions and benzofuran derivatives interactions showed low energies and binding with different residues as reported previously (Sagaama et al., 2020b; Issa et al., 2020; Sagaama et al., 2021).

Our docking studies corroborated the experimental findings showing the potential mechanism of action (bond length) of chemical 3 with distinct receptors in close proximity (Table 1). Figure 3 depicts the comprehensive 3D interactions of compound 3. These four proteins were seen to be involved in various pathways so are linked to variable diseases so these were selected for analysis. The increase and decrease of these enzymes was found in many pathways. Experimental analysis was done on these proteins so they were also subjected to computational analysis so that an in-vivo and *in-vitro* link can be obtained.

### 3.3 X-ray crystal structures

**FIGURE 4:**
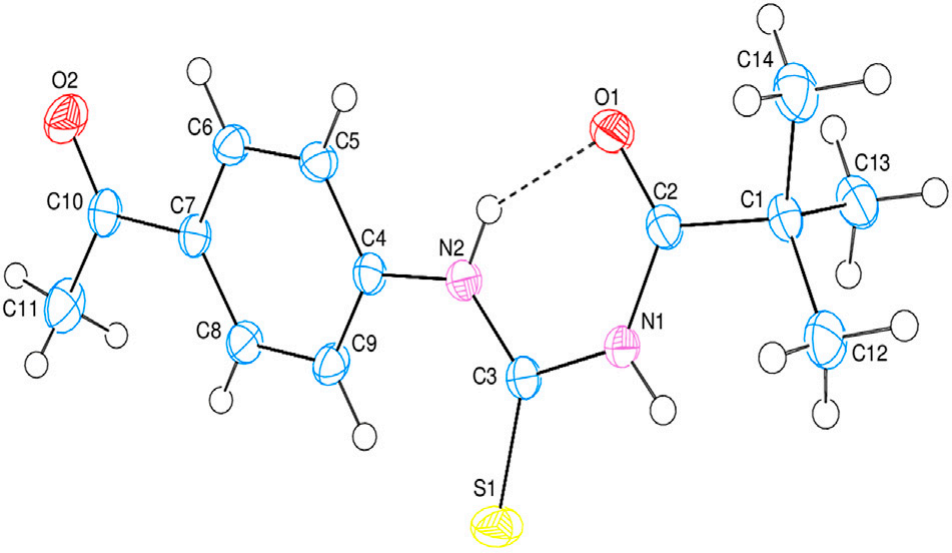
The 3D diagram of title compound with atom numbering scheme.

The intramolecular N—H•••O hydrogen bond generates a S 6) ring motif in the title molecule (Figure 4). N—H•••O interactions exist in crystal structure between the centroids of neighboring parallel aromatic (C4-C9) rings with a centroid to centroid distance of 3.9766 (7) Å [Cg1...Cg1i = 3.9766 (7Å)], where Cg1 is the A (C4-C9) ring centroid [Symmetry code: 1) –x, –y, –z] in which they may be useful for structural stabilization. In the structure, there is no C—H•••π interaction was observed.

### 3.4 Hirshfeld surface analysis

**FIGURE 5:**
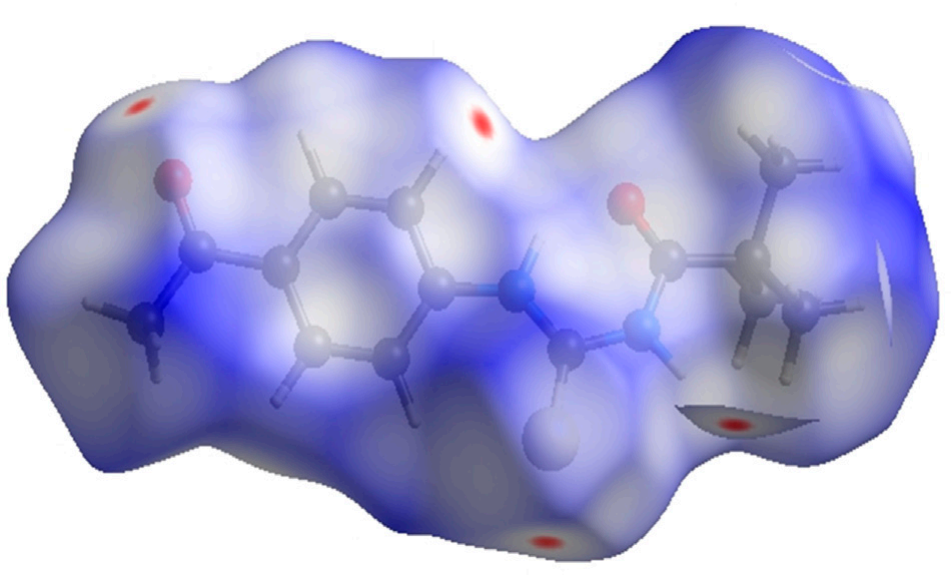
3D diagram of title compound plotted over d_norm_ with range (-0.0688 to 1.3807 a. u).

**FIGURE 6:**
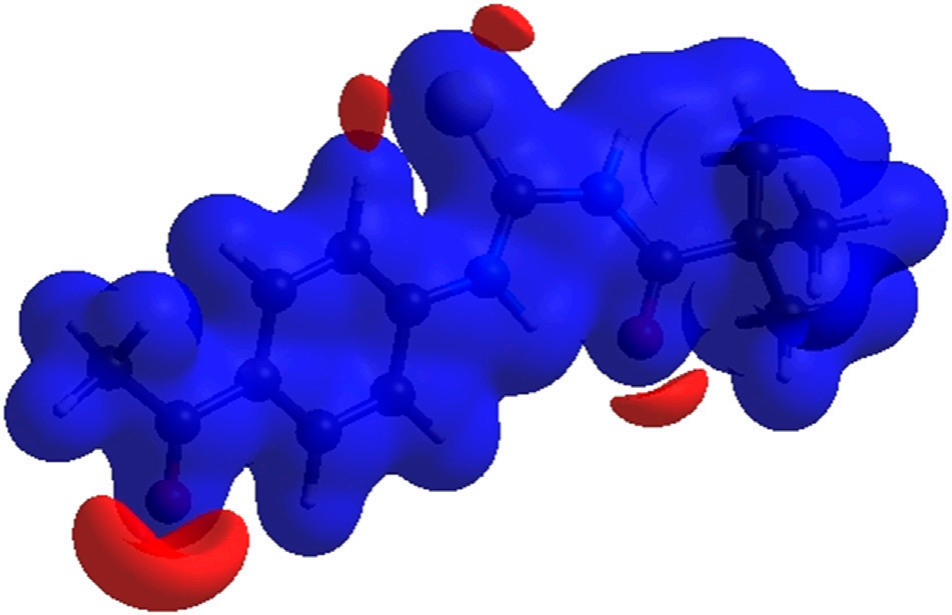
3D diagram of title compound determined by electrostatic potential energy using the STO-3 G basis set at the Hartree–Fock level of theory. Hydrogen-bond donors and acceptors are shown as blue and red regions presenet around the atoms indicate hydrogn-bond donors and acceptors positive and negative potential.

**FIGURE 7:**
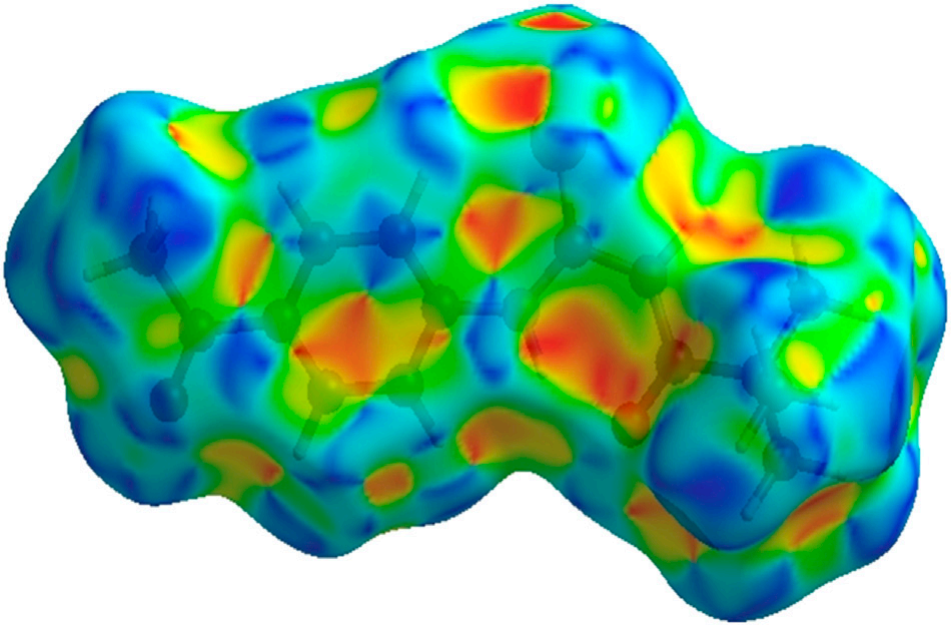
3D diagram of title compound determined by using shape-index.

**FIGURE 8:**
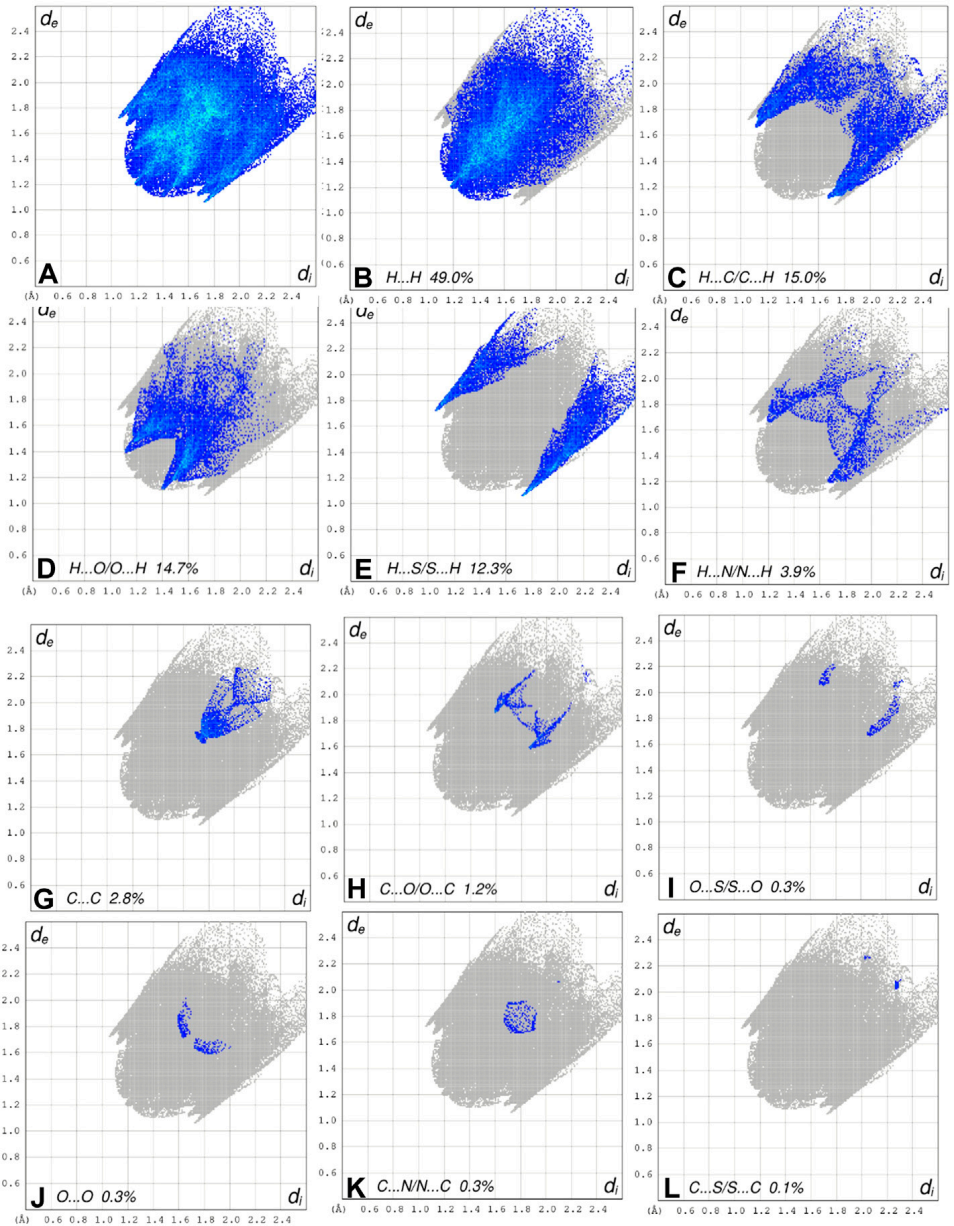
The 2D fingerprint plots of the title compound, showing **(A)** all interactions, and delineated into **(B)** H ••• H, **(C)** H ••• C/C ••• H, **(D)** H ••• O/O ••• H, **(E)** H ••• S/S••• H, **(F)** H ••• N/N ••• H, **(G)** C ••• C/, **(H)** C ••• O/O ••• C, **(I)** O ••• S/S ••• O, **(J)** O ••• O interactions, **(K)** C •••N/N •••C and **(L)** C ••• S/S •••C

**FIGURE 9:**
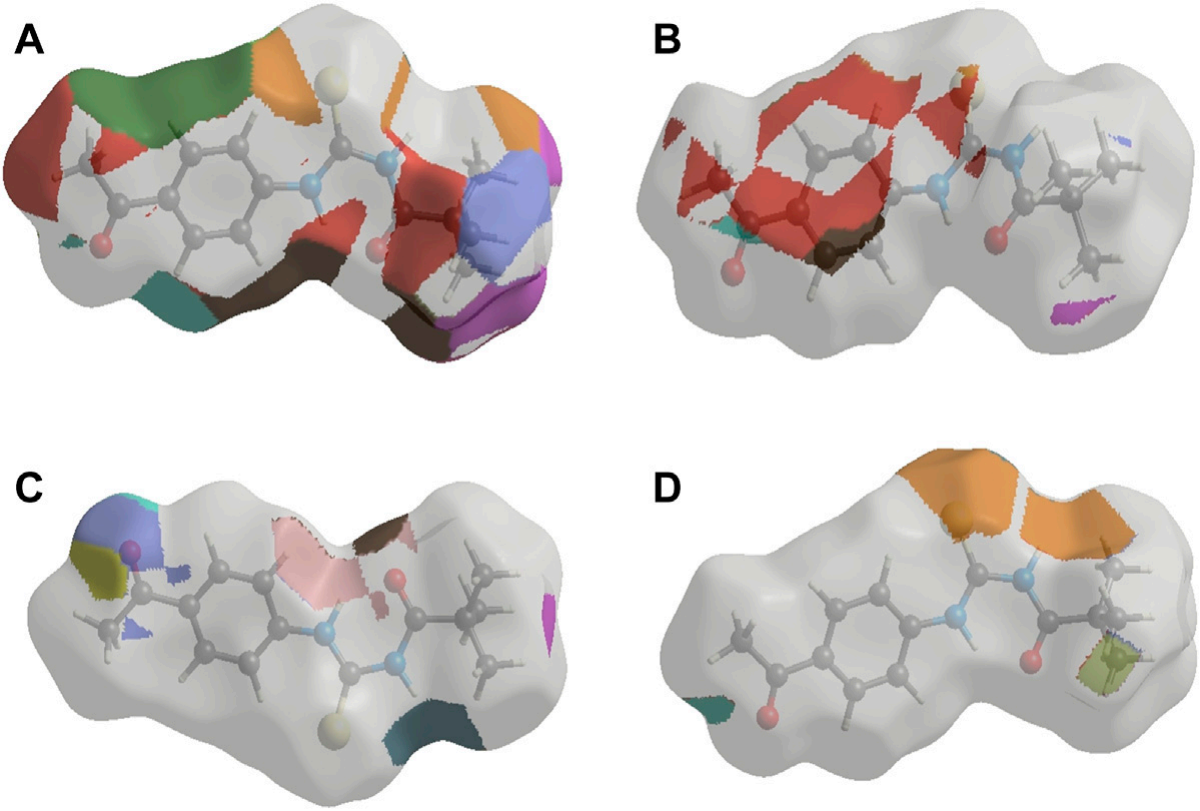
The 3D representations of Hirshfeld surface with the function d_norm_. **(A)** H ••• H, **(B)** H ••• S/S ••• H, **(C)** H ••• C/C ••• H and **(D)** H ••• O/O••• H interactions.

**FIGURE 10:**
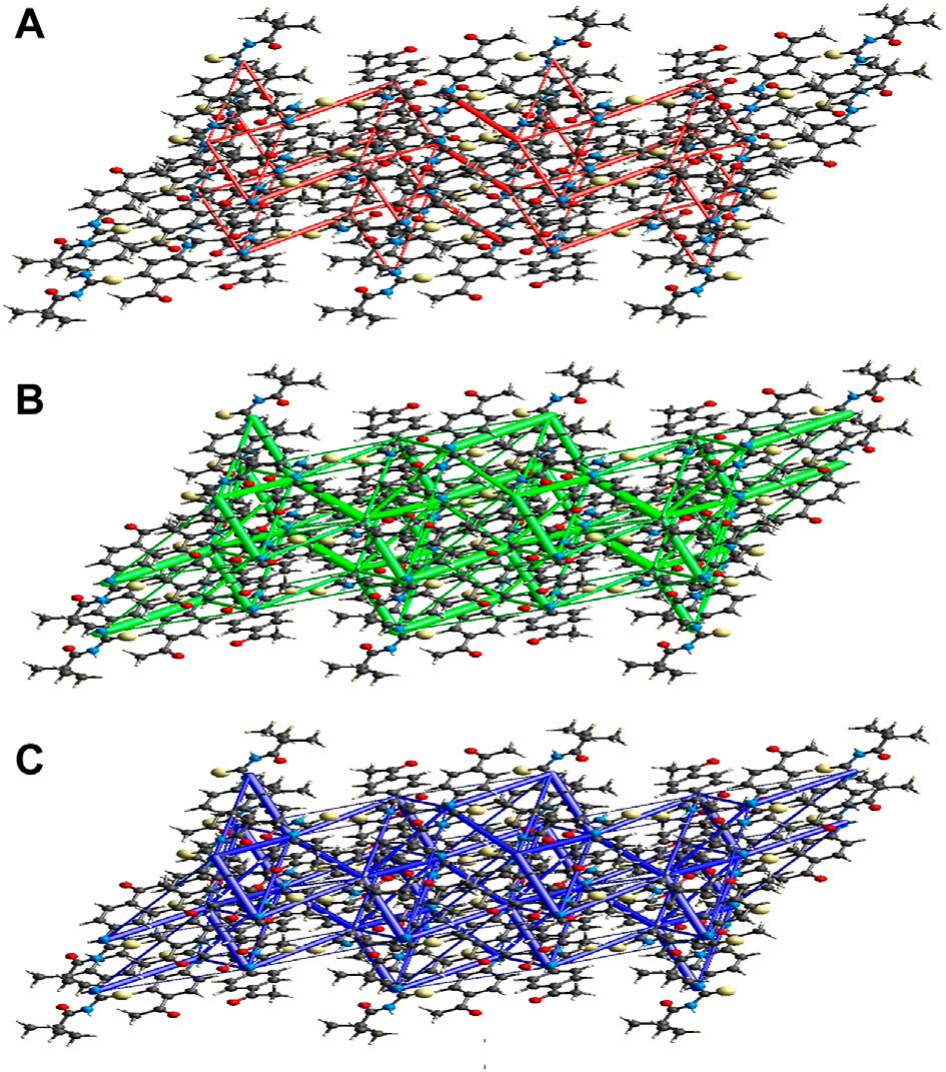
Perspective views of the energy frameworks for a cluster of the title compound molecules showing different energy forms i.e., **(A)** electrostatic energy, **(B)** dispersion energy and **(C)** total energy diagrams.

Crystal Explorer 17.5 was used to conduct a Hirshfeld surface (HS) investigation to visualize the intermolecular interactions in the crystal of the title chemical (3). The outcomes of the HS analysis are shown in the figures below (Figures 5,6,7,8,9,10). All other pertinent information, including experimental details and the complete methodology for HS analysis, is included in the supplementary file (Supplementary Tables 1,2).

#### 3.4.1 Interaction energy calculations and energy frameworks

The intermolecular interaction energies are calculated using the CE–B3LYP/6–31G (d,p) energy model in Crystal Explorer 17.5, which generates a cluster of molecules by applying crystallographic symmetry operations with respect to a central molecule within a radius of 3.8 by default. The sum of electrostatic (E_ele_), polarisation (E_pol_), dispersion (E_dis_), and exchange-repulsion (E_rep_) energies with scale factors of 1.057, 0.740, 0.871, and 0.618, respectively, is the total intermolecular energy (E_tot_).

The calculation of intermolecular interaction energies is combined with a graphical representation of their magnitude in energy frameworks. The relative strength of the respective interaction energy is represented by cylinders connecting the centroids of pairs of molecules, with the cylinder radius proportional to it. E_ele_ (red cylinders), E_dis_ (green cylinders), and E_tot_ (blue cylinders) each have their own energy system (Figure 10A, b and c). The electrostatic, dispersion, and total energy frameworks all show that the dispersion energy contribution dominates the stabilization in the title compound.

### 3.5 Density functional theory studies

**FIGURE 11:**
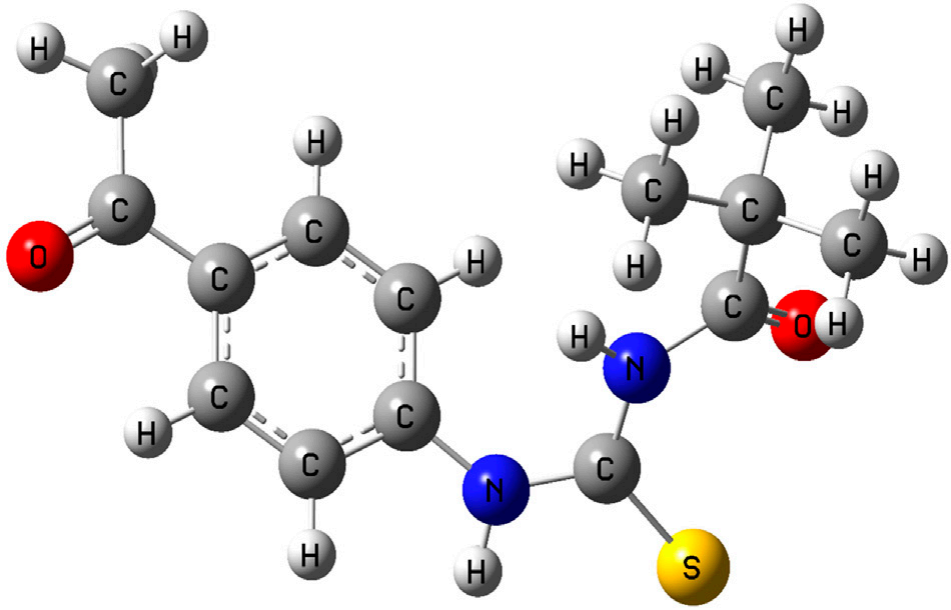
Optimized geometry of compound 3.

The shape of the compound has been tuned for the steepest energy gradient. No illusionary frequency was detected, proving that the optimised structure is a genuine local minimum. The optimised structure is seen in Figure 11.

The chemical composition of a drug and its interaction with a target protein are largely determined by its molecular stability and reactivity. FMO analysis provides sufficient information regarding the compound’s reactivity. HOMO is more likely to donate an electron, while LUMO is more likely to accept it. The energy gap between HOMO and LUMO controls the compound’s chemical reactivity. A compound with a small energy gap is more reactive than one with a big energy gap, which tends to be a more stable molecule. The modest energy gap of 0.1284 eV exhibited by chemical 3 demonstrates its reactive nature. Softness and hardness are crucial factors in determining the chemical reactivity of a molecule. Compound 3 was reported to have a high score for chemical softness, 7.785η. A substance with a high value for chemical softness is highly reactive. Table 2 provides the HOMO/LUMO energy gap and hardness softness values for selected compounds.

**TABLE 2:** Geometric parameters of the compound 3.

Compound	Optimization Energy (hartree)	Polarizability a.u (α)	Dipole Moment (Debye)	E_HOMO_ (eV)	E_LUMO_ (eV)	∆E_gap_ (eV)	Softness (η)	Hardness (η)
3	−1,202.25	196.98	5.23	−0.211	−0.08	0.12	7.78	0.06

**FIGURE 12:**
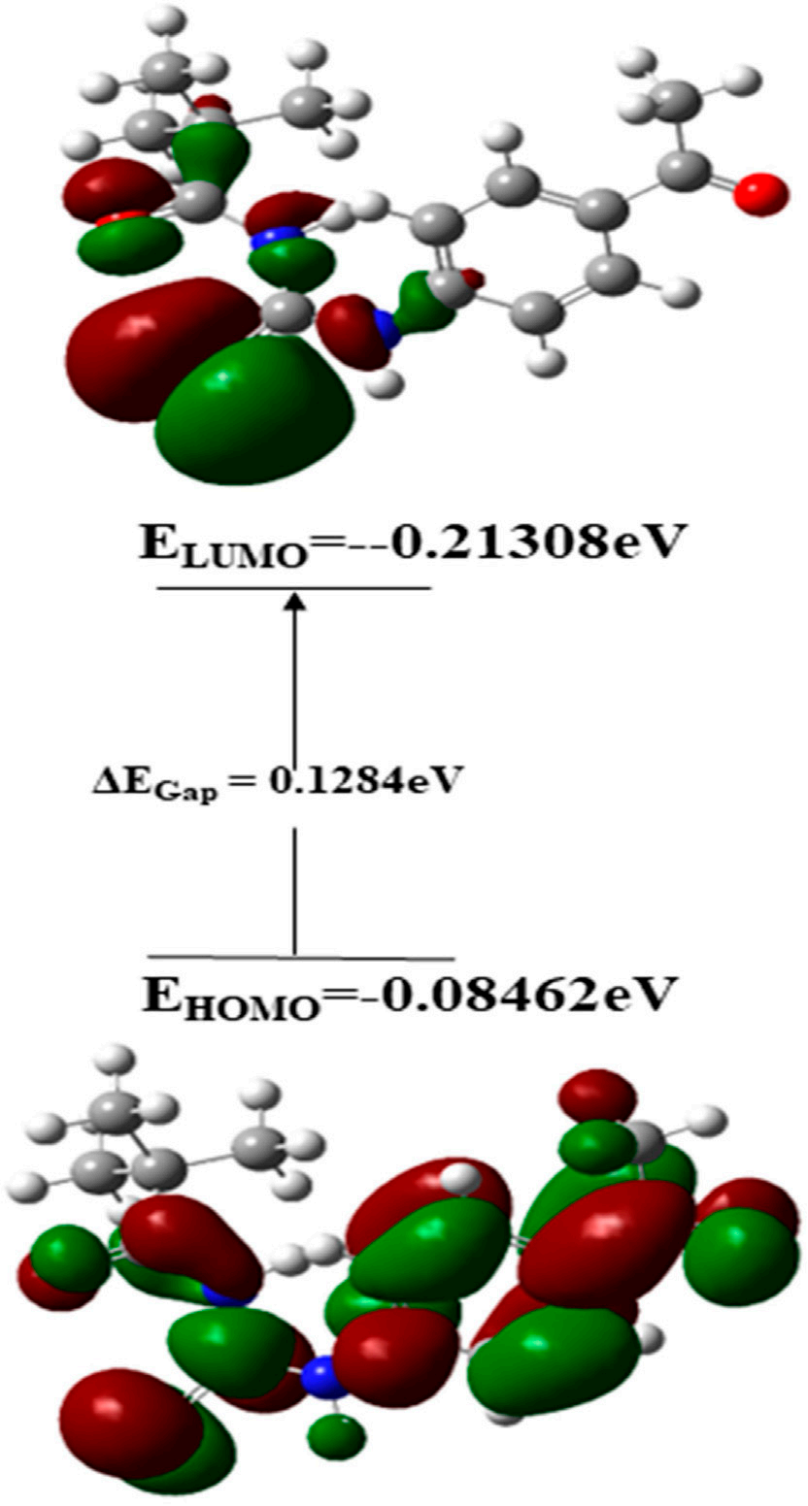
Frontier molecular orbitals of compound 3.

In terms of Frontier molecular orbital analysis, it was observed that HOMO orbitals were majorly confined to pyrimidine ring of the compound which demonstrate that this part was majorly responsible for chemical reactivity of the compound 3. Whereas, LUMO orbital was confined to phenyl ring of the compound 3 which might act as electron acceptor. FMO orbitals are shown in Figure 12.

## 4 Conclusion

In the current study, *N*-((4-acetyl phenyl) carbamothioyl) pivalamide was synthesized and characterized by using spectroscopic and single crystal assays. DFT analysis revealed the reactive nature of the compound with a narrow LUMO/HOMO energy gap. The narrow energy gap between Frontier orbitals is predicting the reactive nature of (3). Hirshfeld analysis provided significant insight into intermolecular and non-covalent interactions existed among crystal structure. According to Hirshfeld surface analysis of the crystal structure the most substantial contributions to the crystal packing are from H ••• O and H ••• N/N ••• H interactions. X-ray refinement data demonstrated that C—H•••O and N—H•••O contacts stabilized molecule geometry, whereas hydrophobic interactions including van der Waal forces also stabilized the crystal packing. The *in-vitro* enzyme inhibition assay was conducted to evaluate the multi-targeting nature of title molecule. The crystal exhibited approximately 85% enzyme inhibition activity against BChE and AChE and 73.8 % and 57.9% inhibition activity against urease and alpha amylase respectively. Moreover, *in-silico* binding orientation of crystal was assessed through molecular docking approach. It was observed that compound 3 produced stable molecular interactions against all targeted enzymes particularly AChE and BChE with docking score of −7.5 and −7.6 kcal/mol respectively. Moreover, the electrostatic surface potential map reveals that atoms N1 and N2 are electron donors, whereas atoms O1 and O2 are electron acceptors. On the basis of the results, it was determined that compound 3 featured a reactive profile and powerful anti-enzyme activity, making it a potential new therapeutic strategy against AChE and BChE-associated malignancies.

## Data Availability

Dataset associated with present study can be found at publicly accessible structural database. Crystallographic data for the structure reported herein have been deposited with the Cambridge Crystallographic Data Centre as Supporting Information. The dataset can be found at following link: http://www.ccdc.cam.ac.uk/2176055
