# Supplementary material for: Facile synthesis, crystal structure, biological evaluation, and molecular modeling studies of N-((4-acetyl phenyl) carbamothioyl) pivalamide as the multitarget-directed ligand
Source: Front Chem. 2022 Sep 26;10:992701. doi: 10.3389/fchem.2022.992701 (PMC9549587; doi:10.3389/fchem.2022.992701)
Supplement: Supplementary file 1 [file DataSheet1.docx]

Supplementary Material

**Facile Synthesis, Crystal Structure, Biological Evaluation and Molecular Modeling Studies of *N*-((4-acetyl phenyl) carbamothioyl) pivalamide as the multitarget-directed ligand**

Aamer Saeed^1^*, Syeda Abida Ejaz^2^*, Aqsa Khalid^1^, Pervaiz Ali Channar^1,3^ Mubashir Aziz^2^, Tanveer A. Wani^4^, Seema Zargar^5^, Sidra Hassan^6^, Hammad Ismail^7^, Dania Khalid^7^, Muhammad Zaffar Hashmi^8^, Tuncer Hökelek^9^, Abdullahi Tunde Aborode^10^

*^1^Department of Chemistry, Quaid-i-Azam University-45320 Islamabad, Pakistan*

*^2^Department of Pharmaceutical Chemistry, faculty of Pharmacy, The Islamia University of Bahawalpur, Pakistan.*

*^3^Department of Basic Sciences, Mathematics and Humanities, Dawood University of*

*Engineering and Technology, Karachi 74800, Pakistan.*

*^4^Department of Pharmaceutical Chemistry, College of Pharmacy, King Saud University, P.O. Box 2457, Riyadh 11451, Saudi Arabia*

*^5^Department of Biochemistry, College of Science, King Saud University, P.O. Box 22452, Riyadh 11451, Saudi Arabia*

^6^Bahawalpur College of Pharmacy, Bahawalpur Medical and Dental College, Bahawalpur, Pakistan.

*^7^Department of Biochemistry and Biotechnology, University of Gujrat, Gujrat, 50700, Pakistan*

*^8^Department of Chemistry, COMSATS University Islamabad, 44000, Pakistan*

*^9^Department of Physics, Faculty of Engineering, Hacettepe University, Beytepe-Ankara, 06800, Turkey*

*^11^Department of Chemistry, Mississippi State University, Starkville, USA*

***Correspondence:**

**Aamer Saeed**

[aamersaeed@yahoo.com](mailto:aamersaeed@yahoo.com);

[asaeed@qau.edu.pk](mailto:asaeed@qau.edu.pk) (<https://orcid.org/0000-0002-7112-9296>)

**Syeda Abida Ejaz**; [abida.ejaz@iub.edu.pk](mailto:abida.ejaz@iub.edu.pk); [abidaejaz2010@gmail.com](mailto:abidaejaz2010@gmail.com)

**X-ray crystal structure and X-ray refinement**

The crystallographic data of synthesized compound 3 was collected by a diffractometer (Rigaku Oxford Diffraction Xcalibur, Eos, Gemini) equipped with Cu *K*_α_ radiation (*λ* = 1.54184 Å For solving and refining the structure, the multi-scan absorption correction (*CrysAlis PRO* 1.171.38.46) applied data were processed by two SHELX program packages, SHELXT and SHELXL,while for drawing, the ORTEP-3 and PLATON programs were used. The positions of hydrogen atoms were geometrically evaluated as 0.88 Å for NH, 0.95 Å for CH and 0.98 Å for CH_3_. A riding model with the limitations of U_iso_(H) = k X U_eq_ (C, N) was used for refining, where k = 1.2 for NH and CH hydrogens and k =1.5 for CH_3_ hydrogens. Crystallographic data for the structure reported herein have been deposited with the Cambridge Crystallographic Data Centre as Supporting Information, CCDC No. 2176055. Copies of the data can be obtained through application to CCDC, 12 Union Road, Cambridge CB2 1EZ, UK. (fax: +44 1223 336033 or email: [deposit@ccdc.cam.ac.uk](mailto:deposit@ccdc.cam.ac.uk) or at <http://www.ccdc.cam.ac.uk>).

**Hirshfield Analysis**

**Supplementary table 1.** Hydrogen-bond geometry (Å, º).

| *D*—H···*A* | *D*—H | H···*A* | *D*···*A* | *D*—H···*A* |
| --- | --- | --- | --- | --- |
| N2—H2···O1 | 0.88 | 1.85 | 2.5996 (13) | 142 |

**Supplementary table 2.** Selected interatomic distances (Å).

| S1···C9 | 3.2301 (13) | C2···H2 | 2.38 |
| --- | --- | --- | --- |
| S1···H9 | 2.62 | C8···H11B | 2.85 |
| S1···H12C^i^ | 2.98 | C11···H8 | 2.65 |
| S1···H13A^i^ | 2.90 | C12···H1 | 2.42 |
| O1···H14A | 2.48 | C14···H8^iii^ | 2.90 |
| O1···H2 | 1.87 | H1···H12C | 2.04 |
| O2···H6 | 2.47 | H2···H5 | 2.22 |
| O2···H5^ii^ | 2.61 | H8···H11B | 2.34 |
| N1···H12C | 2.68 |  |  |

NMR and FTIR data


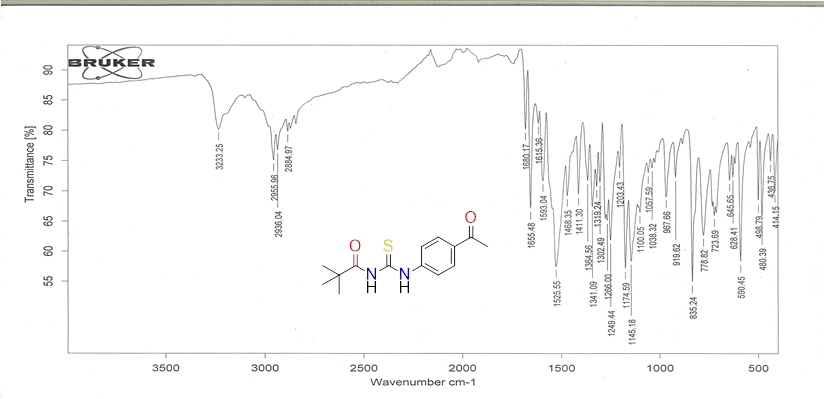

​
